# Population based trends in mortality, morbidity and treatment for very preterm- and very low birth weight infants over 12 years

**DOI:** 10.1186/1471-2431-12-17

**Published:** 2012-02-22

**Authors:** Christoph Rüegger, Markus Hegglin, Mark Adams, Hans Ulrich Bucher

**Affiliations:** 1 Division of Neonatology, University Hospital Zurich, Zurich, Switzerland; 2 Division of General Pediatrics, Graubuenden Cantonal Hospital, Chur, Switzerland

## Abstract

**Background:**

Over the last two decades, improvements in medical care have been associated with a significant increase and better outcome of very preterm (VP, < 32 completed gestational weeks) and very low birth weight (VLBW, < 1500 g) infants. Only a few publications analyse changes of their short-term outcome in a geographically defined area over more than 10 years. We therefore aimed to investigate the net change of VP- and VLBW infants leaving the hospital without major complications.

**Methods:**

Our population-based observational cohort study used the Minimal Neonatal Data Set, a database maintained by the Swiss Society of Neonatology including information of all VP- and VLBW infants. Perinatal characteristics, mortality and morbidity rates and the survival free of major complications were analysed and their temporal trends evaluated.

**Results:**

In 1996, 2000, 2004, and 2008, a total number of 3090 infants were enrolled in the Network Database. At the same time the rate of VP- and VLBW neonates increased significantly from 0.87% in 1996 to 1.10% in 2008 (p < 0.001). The overall mortality remained stable by 13%, but the survival free of major complications increased from 66.9% to 71.7% (p < 0.01). The percentage of infants getting a full course of antenatal corticosteroids increased from 67.7% in 1996 to 91.4% in 2008 (p < 0.001). Surfactant was given more frequently (24.8% in 1996 compared to 40.1% in 2008, p < 0.001) and the frequency of mechanical ventilation remained stable by about 43%. However, the use of CPAP therapy increased considerably from 43% to 73.2% (p < 0.001). Some of the typical neonatal pathologies like bronchopulmonary dysplasia, necrotising enterocolitis and intraventricular haemorrhage decreased significantly (p ≤ 0.02) whereas others like patent ductus arteriosus and respiratory distress syndrome increased (p < 0.001).

**Conclusions:**

Over the 12-year observation period, the number of VP- and VLBW infants increased significantly. An unchanged overall mortality rate and an increase of survivors free of major complication resulted in a considerable net gain in infants with potentially good outcome.

## Background

Very preterm birth is a major cause of mortality and morbidity for newborns and imposes a considerable burden on limited health care resources. Over the last two decades, changes in perinatal management have been associated with a significant increase and better outcome of these infants [1,2]. However, the majority of these reports are based on single centres or neonatal networks not representing the whole population. In addition data may be biased by different criteria for referral, admission or treatment [3]. Only a few publications analyse the short-term outcome of these infants on a nationwide basis over more than ten years. On these grounds, the Swiss data from 1996, 2000, 2004, and 2008 were analysed, focussing on temporal trends in mortality, morbidity and treatment for VP- and VLBW infants. Special importance was attached to the short-term survival free of major complications. Beyond that, temporal changes in the length of hospital stay as a substitute for the resources needed were followed. These results were finally compared with studies in other countries. Referring to previous population-based studies, we hypothesised that improvement in obstetric and perinatal management led to a decrease in mortality resulting in more survivors with disability.

## Methods

The Swiss Neonatal Network & Follow-Up Group, a non-profit voluntary collaboration of health care professionals was founded by the Swiss society of Neonatology in 1995 with the goal to improve the quality of neonatal care. Today, the Network comprises all nine Neonatal Intensive Care Units (NICUs), most of the smaller Neonatal Units (NUs) and most Neuropediatric Centres caring for VP and VLBW infants in Switzerland under the auspices of the Swiss Society of Neonatology. The Network maintains a Minimal Neonatal Data Set (MNDS) collecting anonymous information about the demographics and outcome of all liveborn infants between 400 and 1500 g birth weight and/or between 23 0/7 and 31 6/7 gestational weeks, born at or transferred to a participating hospital. Data were collected on all infants until death or discharge home. Mortality rates were calculated for all infants born alive. Morbidity rates and treatments however were based only on those infants admitted to a NICU, and encompass the following diagnoses: intraventricular haemorrhage (IVH), based on the most severe ultrasound result during the hospital stay using the classifications defined by Papile et al. [4]; cystic periventricular leucomalacia (PVL) defined by de Vries et al. [5]; retinopathy of prematurity (ROP) using the international classification published by the committee for the classification of ROP [6]; bronchopulmonary dysplasia (BPD) defined as an oxygen requirement at 36 weeks gestational age (GA) according to the NICHD consensus conference paper [7]; necrotising enterocolitis (NEC) defined as clinical signs (abdominal distension, bilious aspirates and/or bloody stools) confirmed by radiographically visible intramural gas or at laparotomy (Bell stage 2 and 3) [8]; patent ductus arteriosus (PDA) which was symptomatic and required indomethacin or surgery; sepsis with clear clinical, radiological, or histological evidence of infection as well as at least one microbiologically relevant positive blood culture. A survival free of major complications was determined as survival without grade 3 and 4 IVH, cystic PVL, ROP stage 3 or 4 or BPD. The years 1996, 2000, 2004 and 2008 were chosen because the Swiss Neonatal Network and Follow-up Group made a special effort to ensure that data of these years were complete and correct. To assess the completeness of our data, the number of infants having been enrolled since 1996 were compared to the birth registry of the Swiss Federal Statistical Office [9]. Data were collected for 89% of all VLBW infants in 1996, 90% in 2000, 97% in 2004 and 90% in 2008.

### Statistical analysis

A two-sided paired Student's *t*-test was performed to compare mean values of two independent, normally distributed variables. To determine temporal changes in the distribution of a variable, the Pearson's Chi-square test was used. Probability levels below 0.05 were considered significant. To determine a temporal trend we used linear regression models with the coefficient β indicating the slope of a linear regression line. All statistical analyses were carried out with R release 2.13.0.

## Results

### Demographics

According to the Swiss National Registry, there were 83'007 liveborn babies in 1996, 78'458 in 2000, 73'082 in 2004, and 76'691 in 2008. Concurrently the rates of VLBW infants in Switzerland increased significantly from 0.76% to 0.97% (p_96-08 _< 0.001, β = 0.06%). 3090 infants with less than 32 completed gestational weeks and/or with a birth weight less than 1500 g were included for further analysis. For the demographic details of the study population and their changes over the years see Table 1.

**Table 1:** Demographic changes of the study population from 1996 to 2008

Year	1996-2008	1996		2000		2004		2008	
Characteristics	No. (%)		p-value 1996-2000	No. (%)	p-value 2000-2004	No. (%)	p-value 2004-2008	No. (%)	p-value 1996-2008
Very preterm infants^1^	2665 (86.2)	606 (84.2)	0.07	674 (87.1)	0.24	662 (87.8)	0.04	723 (85.9)	0.23
Very low birth weight infants^2^	425 (13.8)	114 (15.8)	0.03	100 (12.9)	0.57	92 (12.2)	0.09	119 (14.1)	0.18
Small for gestational age^3^	576 (18.6)	146 (20.3)	0.06	136 (17.6)	0.90	134 (17.8)	0.36	460 (19.0)	0.35
Gender									
- female	1507 (48.8)	342 (47.5)	0.11	390 (50.4)	0.22	363 (48.1)	0.65	412 (48.9)	0.41
- male	1583 (51.2)	378 (52.5)		384 (49.6)		391 (51.9)		430 (51.1)	
Location of birth									
- inborn^4^	2806 (90.8)	622 (86.4)	0.07	686 (88.6)	< 0.001	711 (94.3)	0.30	787 (93.5)	< 0.001
- outborn	284 (9.2)	98 (13.6)		88 (11.4)		43 (5.7)		55 (6.5)	
Mode of delivery^5^									
- spontaneous	567 (18.3)	174 (24.2)	< 0.001	143 (18.5)	0.24	127 (16.8)	0.09	123 (14.6)	< 0.001
- caesarean section	2395 (77.5)	521 (72.4)	< 0.01	595 (76.9)	< 0.01	610 (80.9)	0.29	669 (79.5)	< 0.001
Number of infants									
- singleton	2144 (69.4)	541 (75.1)	< 0.01	546 (70.5)	0.13	513 (68.0)	0.03	544 (64.6)	< 0.001
- multiples	946 (30.6)	179 (24.9)		228 (29.5)		241 (32.0)		298 (35.4)	
**Characteristics**	**p0.5 (p0.05-p0.95)**	**Mean**	**p-value 1996-2000**	**Mean**	**p-value 2000-2004**	**Mean**	**p-value 2004-2008**	**Mean**	**p-value 1996-2008**
Gestational age (week)	30 0/7 (25 0/7 - 33 5/7)	29 6/7	< 0.01	29 3/7	1	29 3/7	0.35	29 4/7	0.04
Birth weight (g)	1225 (630 - 1840)	1238	0.13	1209	0.56	1220	0.51	1208	0.11

p0.5 = 50th percentile = median, p0.05 = 5th percentile, p0.95 = 95th percentile, ^1 ^infants born < 32 weeks of gestation, ^2 ^infants born ≥32 weeks of gestation with a birth weight < 1500 g, ^3 ^birth weight < 10th percentile, ^4 ^born in one of the nine perinatal centres, ^5 ^cephalic forceps and cephalic ventouse deliveries were not listed separately

### Mortality

#### Neonatal mortality rate

412 (13.3%) infants died during the study period, 96 (3.1%) of which in the delivery room. We observed 292 (9.4%) early neonatal (perinatal) deaths, defined as a death of a live born child within the first 7 days of life. A late neonatal death, occurring after 7 but before 28 completed days was found in 81 (2.6%) cases. The sum of early and late neonatal deaths amounted to an average of 12.1%. The rates for early-, late-, and neonatal deaths did not change significantly during the 12 years of observation.

#### Survival analysis

**Figure 1:**
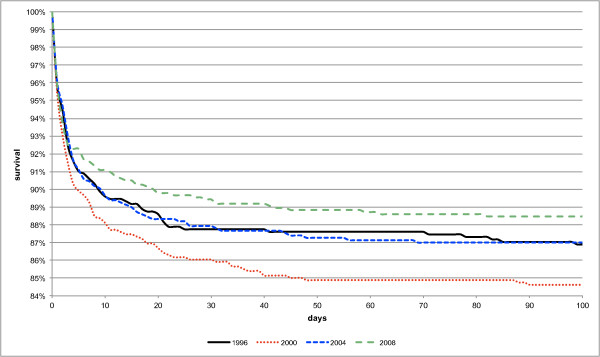
**Kaplan-Meier survival curve per year**.

The survival rate was 86.8% in 1996, 84.1% in 2000, 86.7% in 2004, 88.2% in 2008, and on average 86.5%. The increase from 1996 to 2008 was not significant (p_96-08 _= 0.22, β = 0.70%) even though the Kaplan-Meier analysis (Figure 1) showed an overall better survival in 2008 resulting from considerably higher survival rates during the first 48 days of life. The mean duration till death amounted to 13.4 days in 1996, 12.7 days in 2000, 7.0 days in 2004 and 7.5 days in 2008. During the whole study period only a trend towards a lower mean duration till death was found (p_96-08 _= 0.09, β = -2.3 days).

#### Gestational age

When stratifying the study population according to the GA we could observe significant lower mortality rates in 2008 for the two youngest GA groups (< 26 weeks of gestation: p_96-08 _= 0.02, and 26-27 weeks of gestation: p_96-08 _= 0.04). For the two older GA groups the difference between 1996 and 2008 was not statistically significant (p-values > 0.05). Infants with < 26 completed gestational weeks had a seven times higher relative risk (RR) to die than those who were at least 26 completed gestational weeks old (RR = 6.8). A detailed analysis of survival regarding gender, mode of delivery, location of birth, number of infants, GA and birth weight is given in Table 2.

**Table 2:** Analysis of survival

Year	1996-2008			1996		2000		2004		2008	
Characteristics	No. (%)	**p-value 1. - 2**.	relative risk	No. (%)	p-value 96 - 00	No. (%)	p-value 00 - 04	No. (%)	p-value 04 - 08	No. (%)	p-value 96 - 08
Overall mortality rate	412 (13.3)			95 (13.2)	0.04	122 (15.8)	0.04	98 (13.0)	0.20	97 (11.5)	0.15
Mortality rate by gender											
1. females	177 (11.7)	0.55	1	41 (12.0)	0.26	54 (13.8)	0.07	38 (10.5)	0.91	44 (10.7)	0.41
2. males	235 (14.8)		1.13	54 (14.3)	0.06	68 (17.7)	0.22	60 (15.3)	0.09	53 (12.3)	0.24
Mortality rate by mode of delivery^1^											
1. spontaneous	107 (18.9)	0.17	1.65	33 (19.0)	0.70	29 (20.3)	0.54	23 (18.1)	0.95	22 (17.9)	0.75
2. caesarean section	273 (11.4)		1	58 (11.1)	< 0.01	86 (14.5)	0.03	70 (11.5)	0.03	59 (8.8)	0.06
Mortality rate by location of birth											
1. inborn^2^	376 (13.4)	0.89	1.06	81 (13.0)	0.01	112 (16.3)	0.02	92 (12.9)	0.26	91 (11.6)	0.23
2. outborn	36 (12.7)		1	14 (14.3)	0.43	10 (11.4)	0.60	6 (14.0)	0.51	6 (10.9)	0.47
Mortality rate by number of infants											
1. singleton	308 (14.4)	0.50	1.31	75 (13.9)	0.17	87 (15.9)	0.30	73 (14.2)	0.60	73 (13.4)	0.75
2. multiples	104 (11.0)		1	20 (11.2)	0.05	35 (15.4)	0.03	25 (10.4)	0.18	24 (8.1)	0.09
Mortality rate by gestational age^3^											
< 26	186 (56.0)		6.8^a^	40 (58.8)	0.26	60 (64.5)	0.05	45 (54.2)	0.15	41 (46.6)	0.02
26-27	115 (23.1)		4.7^a^	25 (26.0)	0.95	34 (25.8)	0.51	30 (23.3)	0.17	26 (18.4)	0.04
28-29	52 (7.5)		2.0^a^	14 (8.2)	0.69	13 (7.4)	0.83	12 (7.0)	0.92	13 (7.3)	0.50
30-31	46 (4.0)		1.3^a^	11 (4.0)	0.78	10 (3.7)	0.68	9 (3.2)	0.77	16 (5.0)	0.32

**Characteristics**	**p0.5 (p0.05-p0.95)**	**p-value 1. - 2**.		**mean**	**p-value 96 - 00**	**Mean**	**p-value 00 - 04**	**mean**	**p-value 04 - 08**	**mean**	**p-value 96 - 08**

Birth weight (g)											
1. survivors	1225 (630-1840)	< 0.001		1283	0.49	1279	0.94	1264	0.05	1244	0.01
2. deaths	795 (480-1625)			943	0.02	835	0.11	918	0.79	935	0.89
Gestational age (week)											
1. survivors	30 0/7 (25 0/7 - 33 5/7)	< 0.001		30 2/7	0.56	30 0/7	0.96	29 6/7	0.02	29 6/7	< 0.01
2. deaths	26 3/7 (23 6/7 - 31 5/7)			27 2/7	0.10	26 5/7	0.90	26 5/7	0.32	27 0/7	0.52

p0.5 = 50th percentile = median, p0.05 = 5th percentile, p0.95 = 95th percentile, ^1^cephalic forceps and cephalic ventouse deliveries were not listed separately, ^2 ^inborn = born in one oft the nine perinatal centres, ^3 ^children born > 32 completed gestational weeks were not listed separately, ^a ^relative to the older age groups

### Morbidity

The incidence of typical neonatal morbidities and their temporal trends are given in Table 3. This table also analyses these morbidities in combination with other variables, such as gender, birth weight, GA, location of birth, and mode of delivery.

**Table 3:** Incidence of neonatal morbidities and their temporal trends over the years

	BPD^a ^No. (%)	NEC^b ^No. (%)	IVH^c ^No. (%)	PVL^d ^No. (%)	PDA^e ^No. (%)	RDS^f ^No. (%)	ROP^g ^No. (%)	Sepsis No. (%)
**1996 - 2008^1 ^**(n = 2983)	470 (15.7)	76 (2.5)	176 (5.9)	66 (2.2)	584 (19.6)	2428 (81.4)	38 (1.2)	291 (9.8)
1996^1 ^(n = 702)	125 (17.8)	23 (3.3)	43 (6.1)	12 (1.7)	105 (15.0)	550 (78.3)	13 (1.9)	60 (8.5)
2000^1 ^(n = 750)	104 (13.9)	22 (2.9)	51 (6.8)	18 (2.4)	128 (17.1)	584 (77.9)	4 (0.5)	81 (10.8)
2004^1 ^(n = 728)	123 (16.9)	17 (2.3)	49 (6.7)	21 (2.9)	148 (20.3)	615 (84.5)	9 (1.2)	65 (8.9)
2008^1 ^(n = 803)	118 (14.7)	14 (1.7)	33 (4.1)	15 (1.9)	203 (25.3)	679 (84.6)	12 (1.5)	85 (10.6)

**p-value 1996-2008**	0.02	0.01	0.02	0.71	< 0.001	< 0.001	0.40	0.03

Gender								
- female	217 (14.8)	42 (2.9)	75 (5.1)	39 (2.7)	300 (20.5)	1156 (79.1)	14 (1.0)	130 (8.9)
- male	251 (16.5)	34 (2.2)	100 (6.6)	27 (1.8)	284 (18.7)	1272 (83.6)	22 (1.4)	161 (10.6)
Location of birth								
- inborn^2^	417 (15.5)	65 (2.3)	149 (5.5)	59 (2.2)	529 (19.6)	2205 (81.7)	30 (1.1)	267 (9.9)
- outborn	51 (18.0)	11 (3.9)	26 (9.2)	7 (2.5)	55 (19.4)	223 (78.5)	6 (2.1)	24 (8.5)
Mode of delivery^3^								
- spontaneous	100 (19.0)	11 (2.1)	42 (8.0)	10 (1.9)	105 (19.9)	428 (81.2)	13 (2.5)	51 (9.7)
- caesarean section	353 (15.0)	63 (2.7)	118 (5.0)	53 (2.3)	450 (19.2)	1914 (81.5)	21 (0.9)	234 (10.0)
Number of infants								
- singleton	365 (17.7)	59 (2.9)	129 (6.2)	53 (2.6)	426 (20.6)	1715 (83.1)	32 (1.5)	216 (10.5)
- multiples	103 (11.2)	17 (1.9)	46 (5.0)	13 (1.4)	158 (17.2)	713 (77.7)	4 (0.4)	75 (8.2)
Gestational age^4^								
< 26	103 (38.7)	16 (6.0)	46 (17.3)	10 (3.8)	111 (41.7)	249 (93.6)	20 (7.5)	55 (20.7)
26-27	180 (37.0)	17 (3.5)	59 (12.1)	13 (2.7)	183 (37.7)	466 (95.9)	9 (1.9)	98 (20.2)
28-29	123 (18.0)	18 (2.6)	48 (7.0)	16 (2.3)	160 (23.4)	624 (91.1)	6 (0.9)	80 (11.7)
30-31	50 (4.4)	18 (1.6)	21 (1.9)	22 (2.0)	113 (10.0)	889 (79.0)	1 (0.1)	42 (3.7)

^1 ^infants who died in the delivery room were excluded, ^2 ^born in one of the nine perinatal centres, ^3 ^cephalic forceps and cephalic ventouse deliveries were not listed separately, ^4 ^children born > 32 completed gestational weeks were not listed separately, ^a ^bronchopulmonary dysplasia, ^b ^necrotising enterocolitis, ^c ^intraventricular haemorrhage grade III or IV, ^d ^cystic periventricular leucomalacia, ^e ^patent ductus arteriosus, ^f ^respiratory distress syndrome, ^g ^retinopathy of prematurity grade 3 or 4

#### Neonatal outcome

**Figure 2:**
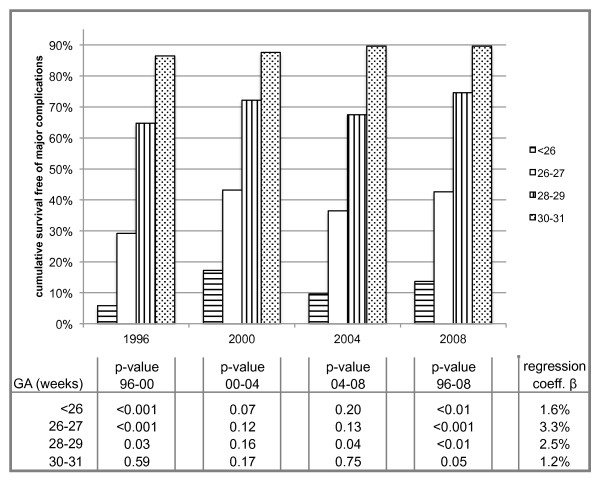
**Gestational age stratified survival free of major complications**.

The overall survival free of major complications was 68.6%. 66.9% in 1996, 68.0% in 2000, 67.5% in 2004 and 71.7% in 2008, reflecting a significant improvement in the short-term outcome over time (p_96-08 _< 0.01, β = 1.4%). The age-stratified survival free of major complication is evident from Figure 2.

#### Length of stay

**Figure 3:**
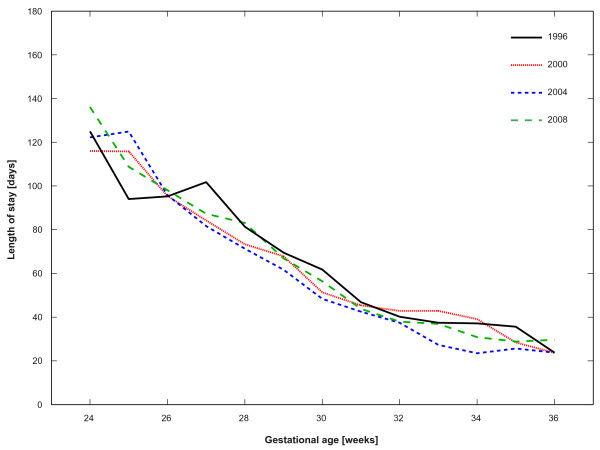
**Gestational age stratified length of stay**.

The mean length of stay (LOS) was based upon the survivors only and amounted to 59.7 days in 1996, 58.5 days in 2000, 55.0 days in 2004 and 60.1 days in 2008. For the overall study period, an average in-hospital stay of 58.4 days was calculated (p_96-00 _= 0.81, p_00-04 _= 0.17, p_04-08 _< 0.01, p_96-08 _= 0.81, β = -0.2 days). The GA was inversely correlated with the LOS and reached up to 108 days for infants < 26 weeks i.e. 21, 42 and 63 days longer than for infants born between 26-27, 28-29 and 30-31 completed gestational weeks respectively. Between 1996 and 2008 we found a significant increase in the LOS for infants born < 26 gestational weeks (p_96-08 _= 0.01, β = 4.3 days) as well as a significant decrease for infants born between 26-27 gestational weeks (p_96-08 _= 0.04, β = -2.9 days). The age stratified LOS are shown in Figure 3. Male infants and singletons were significantly longer hospitalised than females and multiples (59.6 vs. 57.1 days, p = 0.047 and 60.2 vs. 54.4 days, p < 0.001). Over the twelve years of observation, there were no significant changes in the LOS regarding gender, number of infants, mode of delivery, and location of birth (all p_96-08 _> 0.05).

### Therapies

Information about administration of antenatal steroids, surfactant treatment and oxygen therapy are presented in Table 4.

**Table 4:** Treatment of the liveborns

	supplemental oxygen No. (%)		surfactant No. (%)		antenatal steroids No. (%)	
**1996-2008**^**1**^	1933 (64.8)		972 (32.6)		2311 (77.5)	
1996^1^	472 (67.2)		174 (24.8)		475 (67.7)	
2000^1^	489 (65.2)		221 (29.5)		508 (67.7)	
2004^1^	492 (67.6)		255 (35.0)		594 (81.6)	
2008^1^	480 (59.8)		322 (40.1)		734 (91.4)	

**p-value 1996-2008**	< 0.001		< 0.001		< 0.001	

	**No. (%)**	**p-value**	**No. (%)**	**p-value**	**No. (%)**	**p-value**

Gender						
- female	922 (63.1)	0.77	435 (29.8)	0.50	1132 (77.4)	0.99
- male	1011 (66.5)		537 (35.3)		1179 (77.5)	
Location of birth						
- inborn^2^	1728 (64.0)	0.48	865 (32.0)	0.49	2144 (79.4)	0.08
- outborn	205 (72.2)		107 (37.7)		167 (58.8)	
Mode of delivery^3^						
- spontaneous	327 (62.0)	0.76	141 (26.8)	0.38	392 (74.4)	0.71
- caesarean section	1537 (65.5)		789 (33.6)		1852 (78.9)	
Number of infants						
- singleton	1387 (67.2)	0.49	722 (35.0)	0.49	1525 (73.8)	0.35
- multiples	546 (59.5)		250 (27.2)		786 (85.6)	
Gestational age^4^						
< 26	234 (88.0)		167 (62.8)		201 (75.6)	
26-27	435 (89.5)		286 (58.8)		377 (77.6)	
28-29	534 (78.0)		261 (38.1)		536 (78.2)	
30-31	234 (20.8)		234 (20.8)		903 (80.3)	

^1 ^infants who died in the delivery room were excluded, ^2 ^born in one of the nine perinatal centres, ^3 ^cephalic forceps and cephalic ventouse deliveries were not listed separately, ^4 ^children born > 32 completed gestational weeks were not listed separately

#### CPAP treatment

**Figure 4:**
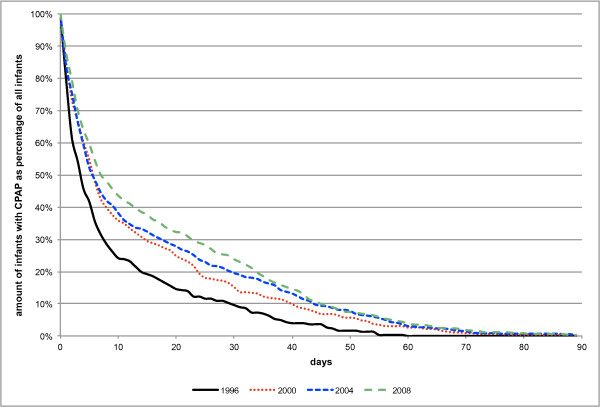
**Cumulative curve of survivors with CPAP treatment per year**.

Continuous positive airway pressure (CPAP) was given to 63.6% of the included infants namely 43.0% in 1996, 60.7% in 2000, 75.8% in 2004 and 73.2% in 2008, resulting in a significant increase of 70.4% between 1996 and 2008 (p_96-08 _< 0.001, β = 10.6%). Most of the newborns who had to be treated with CPAP were those with a GA between 26-27 weeks namely 79.8%, whereas the figures for infants born > 26, 28-29, and 30-31 completed gestational weeks accounted for 65.4%, 76.8% and 60.4% respectively. All GA groups showed a significant shift towards a more frequent use of CPAP therapy (all p_96-08 _< 0.001, β _< 26 GA _= 13.0%, β_26-27 GA _= 8.4%, β_28-29 GA _= 11.5%, β_30-31GA _= 12.4%). This change could most impressively be documented in the age group of infants born between 30-31 gestational weeks. With 36.2% in 1996 and 71.2% in 2008 the incidence of CPAP therapy nearly doubled. There was no difference in the use of CPAP regarding gender (females vs. males, p = 0.78), number of infants (singletons vs. multiples, p = 0.70), mode of delivery (spontaneous vs. caesarean section, p = 0.60) and location of birth (inborn vs. outborn, p = 0.38), but again, the same significant increase of CPAP treatment was found when analysing all four variables separately over time. The overall mean duration of CPAP administration was 9.7 days taking into account the surviving infants only. The respective figures were 3.9 days in 1996, 8.2 days in 2000, 12.8 days in 2004 and 13.8 days in 2008, which is equal to a 3.4 days' increase every four years (p_96-00 _< 0.001, p_00-04 _< 0.001, p_04-08 _= 0.36, p_96-08 _< 0.001). The cumulative percentage of survivors per year treated with CPAP can be seen in Figure 4.

#### Mechanical ventilation

The frequency of infants who were mechanically ventilated was 45.0% in 1996, 39.6% in 2000, 41.8% in 2004 and 45.6% in 2008, which corresponded to an average rate of 43.0%. Altogether we found significant changes between 1996 and 2000 and between 2004 and 2008 (p_96-00 _< 0.01, p_00-04 _= 0.23, p_04-08 _= 0.03, p_96-08 _= 0.74). Mechanical ventilation was inversely correlated with GA: 84.2% of the infants with < 26 completed gestational weeks, 71.2% of those with 26-27 weeks, 51.4% of those with 28-29 weeks and 28.0% of those with 30-31 weeks were ventilated. We found only one significant difference towards a less frequent use of mechanical ventilation regarding infants born < 26 completed gestational weeks (p_96-08 _= 0.045). However there was no difference concerning mechanical ventilation regarding gender (females vs. males, p = 0.50), number of infants (singletons vs. multiples, p = 0.29), mode of delivery (spontaneous vs. caesarean section, p = 0.81), and location of birth (inborn vs. outborn, p = 0.30). The overall mean duration of mechanical ventilation was 3.6 days when taking into account the surviving infants only. With 3.5 days in 1996, 3.1 days in 2000, 3.7 days in 2004 and 4.1 days in 2008 the changes from one observation period to the other as well as over the whole length of the study were not significant (all p-values > 0.05).

## Discussion

Our study shows a considerable net gain in VP- and VLBW infants discharged home without major complications in a stable population over 12 years. This added value is composed of three factors: 1) Increase of the absolute (122) and relative (16.9%) number of VP- and VLBW infants, stable overall mortality rate and higher rate of survivors without major complications (absolute increase 122, relative 25.3%). This finding was not expected and differs in various aspects from previously published results. We will discuss methodological issues and compare the results with those of other studies for the four main topics, population characteristics, mortality, in-hospital morbidity and therapies. Comparison of such global population based trends must be considered with caution as inclusion criteria, definitions of referral, morbidities, treatments, and discharge policies may vary.

### Obstetrics/delivery/birth characteristics

Obstetric management changed with respect to the percentage of mothers who were treated with antenatal corticosteroids as well as with respect to the mode of delivery. This trend probably reflects the improved chance of survival these infants now have, justifying the greater risk to which the mother is exposed to when undergoing surgery compared to natural childbirth. The number of outborn infants decreased significantly, reflecting an on-going trend to centralise high-risk pregnancies in perinatal centres. As a matter of fact, this corresponds to findings of different studies showing that infants who were born in NICUs had lower mortality rates than infants who were transported extrauterinely [10,11]. As expected, the increasing rates of VP- and VLBW infants in our study population correlated with the mean GA but surprisingly not with the mean birth weight. However the surviving infants were both younger and lighter in 2008 than in 1996. The factors underlying these findings are thought to be improvements in obstetric care and a rise in obstetric interventions during pregnancy [12]. With 18.6%, our percentage of small for gestational age infants was much lower compared to those of other studies. Zeitlin et al. for example reported rates between 32.9 and 35.5% based on the EPIPAGE and MOSAIC cohort including VP infants between 1997 and 2003 [13]. This difference was inexplicable as our study population consisted not only of VP- but also of VLBW infants resulting in more infants with birth weights under the 10^th ^percentile.

### Mortality

The overall mortality rate of our study population remained stable over time on an average of 13.3%. Excluding the VLBW infants, a mortality rate of 15.0% was found. Both rates are similar to those reported by other European studies [13-15]. When focussing on our group of infants with < 26 and with 26-27 weeks of GA the mortality rate decreased significantly over time as shown in Table 2. This decline might predominantly be attributable to significant increases of both the administration of antenatal steroids as well as the use of surfactant treatment, resulting in a better survival of the youngest infants. These results were additionally influenced by the publication of the Swiss guidelines on the care of infants born at the limit of viability in 2002, which were likewise followed by a significantly improved survival of extremely preterm infants [16].

Horbar and the members of the Vermont Oxford network discussed potential explanations for the levelling off in the overall mortality during the last two decades [17]: They hypothesised that firstly an inappropriate use of some interventions, such as either an overuse of interventions for infants unlikely to benefit from them, an underuse of potentially beneficial interventions, or a misuse of interventions by inexperienced or unskilled personnel may have resulted in adverse events and secondly, that health professionals as well as families have become more cautious in extending and continuing intensive care treatments for extremely preterm infants. The third argument of the authors, namely having reached the limits of current technology to support preterm infants at gestational ages near the limits of viability, might not be applicable to Switzerland, as recent data show better survival of these infants [18,19]. Extending this study by children born in 2012, as we plan to do, may confirm this finding.

### Morbidity

Regarding the results of other studies examining the relation between antenatal steroids and respiratory distress syndrome (RDS) [20], we expected to find a decrease in the incidence of RDS, which however, increased significantly by 8% to 84.6%. Ersch et al. demonstrated similar findings in their survey of a geographically limited neonatal population [21]. They found that the incidence of RDS in infants admitted to neonatal units doubled over the last 30 years, which was ascribed to the corresponding increase in the rate of caesarean section. We suggest that in our cohort, the above-mentioned constant mortality rate given, the severity of RDS must have been reduced by the increasing antenatal treatment with corticosteroids. The rising survival of the most immature infants and the infants with RDS did not result in an increased number of infants with BPD, which was unexpected and quite contrary to other reports, where an increased survival resulted in more morbidity, mainly BPD [22-24].

In spite of better detection techniques and more ultrasound examinations being routinely made nowadays, the incidence of serious IVH decreased and the rate of cystic PVL remained stable. Again, there might be a positive influence of antenatal corticosteroids on the incidence of IVH and PVL as was shown in the previously mentioned meta-analysis by Crowley [20]. The diagnosis of a patent arterial duct (PDA) was made more frequently over time. This is most likely due to an intensified diagnostic workup, especially by systematic echocardiographs in VP- and VLBW infants. The incidence of necrotising enterocolitis (NEC) decreased significantly to 1.7% mainly due to the preventive administration of probiotics in Switzerland since 2006 [25].

The survival free of major complications which represents besides mortality the most crucial variable defining neonatal outcome, significantly increased. This finding is remarkable as it was neither adversely affected by an increasing number of VP infants nor by better survival rates of the youngest GA groups. Zeitlin et al. investigated the short-term outcome of live births before 32 weeks of gestation in 10 European regions and found large differences in neurologic and respiratory morbidity despite similar standards of living and healthcare provision [26]. They reported rates between 71.2 and 89.7% for a survival without IVH/PVL or BPD raising questions about variability in treatment decisions and population characteristics. Fanaroff et al. defined a survival without major neonatal morbidity as survival without IVH, NEC, and BPD and found a stable rate of 70% between 1995 and 2002 regarding VLBW infants only [23]. Despite stricter criteria including the absence of IVH, cystic PVL, BPD, and ROP, our results are similar to those of other European and American groups.

### Therapies

The increasing antenatal application of corticosteroids as well as the wide use of rescue surfactant therapy led to a change in respiratory support strategy towards early use of nasal CPAP starting in the delivery room and reducing thereby the need for intubation in preterm babies [27,28]. In our study the increased use of antenatal corticosteroids was not associated with a decreased use of surfactant. That was unexpected and could most likely be explained by additional indications for surfactant administration, namely early administration in the delivery room based on risk not on severity of RDS.

We additionally documented an impressive 70% increase in the use of CPAP as well as a far longer duration of CPAP therapy by approximately 10 days between 1996 and 2008. Despite this, the use of mechanical ventilation did not change in terms of period and number of ventilated infants. Taken together, we think that the increasing rates of antenatal corticosteroid- and postnatal surfactant administration, the decreasing use of supplemental oxygen as well as lung-protective ventilation strategies are among the most important factors to explain our lower BPD rate in 2008. This rate of 14.7% is similar to those reported in the two population-based cohort studies EPIPAGE in 1997 (14.4.%) and MOSAIC in 2003 (15.3%) [13].

### Strengths and limitations of the study

Strengths of our study are 1) Prospective definition of inclusion criteria and specific morbidities remaining unchanged over the whole observation period. 2) The recruitment of subjects based on a homogenous, geographically defined population. 3) An observation period of 12-years, 4) a special effort to enhance data quality by on-site visits and matching with the official population statistics, and 5) presenting temporal trends not only of mortality and morbidity but also of therapies used in a large sample of VP- and VLBW infants.

Our study is limited by the lack of information about the long-term outcome of our infants. However, there is an association between major complications and neurodevelopmental outcome. This data is currently being collected and will be reported later.

## Conclusions

Over the 12-year observation period, the number of VP- and VLBW infants increased significantly. An unchanged overall mortality rate and an increase of survivors free of major complication resulted in a considerable net gain in infants with potentially good outcome. This improved short-term outcome was associated with a shorter hospital stay and therefore less cost. Follow-up of this cohort will show whether this benefit will persist.

## Competing interests

The authors declare that they have no competing interests.

## Authors' contributions

CR was responsible for literature review, statistical analyses, interpretation of data, and manuscript preparation. MH contributed to statistical analyses and data preparation. MA was responsible for the data collection and contributed to the statistical analysis and the manuscript. HUB was responsible for conducting the study and participated in the conception, critical revision and approval of the manuscript. All authors read and approved the final manuscript.

## Pre-publication history

The pre-publication history for this paper can be accessed here:

http://www.biomedcentral.com/1471-2431/12/17/prepub
